# Activation of epithelial proliferation induced by Eimeria acervulina infection in the duodenum may be associated with cholesterol metabolism

**DOI:** 10.18632/oncotarget.8490

**Published:** 2016-03-30

**Authors:** Lili Sun, Haibo Dong, Zhenchao Zhang, Jie Liu, Yun Hu, Yingdong Ni, Roland Grossmann, Ruqian Zhao

**Affiliations:** ^1^ Key Laboratory of Animal Physiology and Biochemistry, Ministry of Agriculture, Nanjing Agricultural University, Nanjing 210095, China; ^2^ Department of Veterinary Parasitic Disease, College of Veterinary Medicine, Nanjing Agricultural University, Nanjing 210095, China; ^3^ Department of Functional Genomics and Bioregulation, Institute of Animal Genetics, FLI, Mariensee, Neustadt a Rbg, Germany

**Keywords:** cell proliferation, cholesterol, coccidial infection, small intestine, miRNAs

## Abstract

Cell proliferation in the intestine is commonly occurred during infection and inflammation to replace damaged enterocytes, and cholesterol as an essential constituent of cell membrane, is required for cell proliferation and growth. Here we found that coccidium-challenged (CC) chickens showed severe damages in intestinal structure, a significant increase of cell proliferation, and an activation of genes expression involved in the innate immune response. Compared to control (CON), CC chickens showed a marked decrease of cholesterol (Tch) level in the circulating system, but a significant increase in local duodenum epithelium. Increase of LDLR protein combined with a significant decrease of CYP27A1 protein expression in duodenum epithelium may contribute to intestinal cholesterol accumulation in CC chickens. Moreover, we found miRNAs targeting to CYP27A1 gene participating in post-transcriptional regulation. Hence, these results provide a new insight for the intervention of epithelial proliferation and cholesterol metabolism in the gastrointestinal tracts.

## INTRODUCTION

The intestinal epithelium represents an exquisite model for the study of stem cell biology especially for investigating the relationship between inflammatory disorders and cell proliferation [1]. In small intestine, the duodenum is the main site containing more adenocarcinomas than jejunum and ileum combined possibly due to biliary or pancreatic effluents. Inflammation is generally considered as a beneficial response to host injury and infection in the intestine, and epithelial damage induces a localized repair response characterized by increased division of stem cells at the bottom of crypts to replace damaged epithelial cells [1]. However, chronic inflammation is increasingly recognized as a high risk factor for the development of disease and intestinal cancer [2]. Coccidiosis infection has been known as a good experimental model for the study of inflammatory response in small intestine [3], which can typically cause the intestinal epithelial sloughing, villus tip damage and enteritis [4]. In poultry industry, especially in intensive production systems, coccidiosis infection is one of the most commonly prevalent and economically important diseases caused by the intestinal protozoa Eimeria [5, 6].

Three species of Eimeria most commonly infect in poultry including Eimeria acervulina, Eimeria maxima and Eimeria tenella, which can lead to severe inflammation and damages to different parts of the intestine [7, 8]. It's well documented that the intestinal epithelium and local immune cells are important for protection against colitis and colitis-associated tumorigenesis at different stages of disease development. The pro-inflammatory cytokines such as interleukin-1β (IL-1β) and interleukin-18 (IL-18) molecules induce inflammation and participate in epithelial repair and healing processes via recruitment and activation of immune cells [9]. Epithelial damage also induces a localized repair response characterized by enhancing division of stem cell at the base of crypts to replace the damaged epithelial cells [1]. Cytokines contribute to restoring epithelial barrier integrity by activating controlled proliferation of stem cells located in the crypt base and turnover of damaged epithelial cells, which prevents systemic dispersion of microflora and the induction of exaggerated inflammatory responses. However, chronic inflammatory diseases constitute major risk factors for the formation of neoplastic regions in the intestine particularly in the colon epithelium [10].

Cholesterol is an essential structural component of cell membranes for maintaining both the structural integrity and fluidity, and it also serves as a precursor for the biosynthesis of steroid hormones [11]. The supply of cholesterol is required for cell proliferation and renewal process. In vertebrates, the liver is considered as the major “control center” for maintenance of the systemic cholesterol homeostasis through de novo biosynthesis, clears cholesterol particles from plasma, and reverses cholesterol transport by conversion into bile acids [12]. During the last decade, however, an additional important role of the intestine in keeping cholesterol homeostasis has become apparent, which makes the intestine a potential target for investigating novel antiatherosclerotic strategies [13]. Cell synthesizes cholesterol through a complex process controlled by several factors and enzymes. With respect to enzymes, 3-hydroxy-3-methylglutaryl-CoA reductase (HMGCR) is the rate-limiting enzyme for cholesterol production [14]. On the other hand, cholesterol-7-alphahydroxylase (CYP7A1) and sterol 27-hydroxylase (CYP27A1) mainly participate in the degradation of cholesterol via the classic bile acid biosynthetic pathway in the liver [6]. CYP27A1 can catalyze the oxidative cleavage of the steroid side chain and hydroxylation of cholesterol to 27-hydroxycholesterol (27-HOC) in the most tissues [15, 16]. With respect to cholesterol uptake from the circulating system, LDL receptor (LDLR) and ApoA1 play a vital role in the biological process [17].

Increased proliferation of stem cells at the crypts has been found in the intestine epithelial damage induced by enteritis [1], however, the local cholesterol metabolism and the biologic mechanism have remained uncertain. We hypothesized that paralleled an increase of cell proliferation, local cholesterol metabolism in the intestine would be altered during epithelial cells renewal and turnover process induced by local infection. Therefore, the aim of this study was to evaluate and quantify the level of cholesterol in the circulation and local intestine, and to investigate the mechanism involved in this process.

## RESULTS

### Alterations in epithelial damage and growth performance of CON and CC groups

As shown in Table 1, coccidium-challenged (CC) chickens showed significantly higher lesion score in the duodenum tissue (P < 0.01) compared to control (CON). The number of oocysts in per gram feces (OPG) was higher in CC group (P < 0.01), which was absent in CON chickens. CC chickens showed a tendency to increase of food intake (P = 0.06) and feed conversion ratio (FCR; P = 0.06) compared to CON. However, the mean body weight gain of CC group was less than CON, yet did not reach the statistical significance (P > 0.05). Moreover, there was no difference in liver and spleen weight or their relative weight to body weight between CC and CON chickens (P > 0.1) (Table 1).

**Table 1:** Effect of Eimeria acervulina infection on the Growth performance

	CON	Coccidium	*P* value
BW,32d (g)	1242.92±33.73	1260.45±36.91	0.73
BW,39d (g)	1727.14±58.18	1767.33±50.48	0.61
BWG,32-39d (g)	542.08±38.05	502.73±53.42	0.55
FI,32-39d (g)	1255.42±54.26	1428.64±67.57	0.06
FCR,32-39d	2.37±0.09	2.75±0.17	0.06
Organs index			
Liver (g)	45.00±1.64	47.67±1.61	0.26
Spleen (g)	2.12±0.12	1.95±0.12	0.32
Liver/BW %	2.61±0.07	2.70±0.07	0.37
Spleen/BW %	0.12±0.01	0.12±0.01	0.94
Other index			
Intestine lesion	0.00±0.00	1.47±0.17	0.00
OPG (g)	0.00±0.00	4.55±0.14	0.00

**Note:** BW: body weight; BWG: body weight gain; FI: feed intake; FCR: feed conversation ratio; OPG: oocysts per gramme. Values are means ± SEM. n =14/group. Data are presented as means ± SEM.

### Histological characteristics

**Figure 1:**
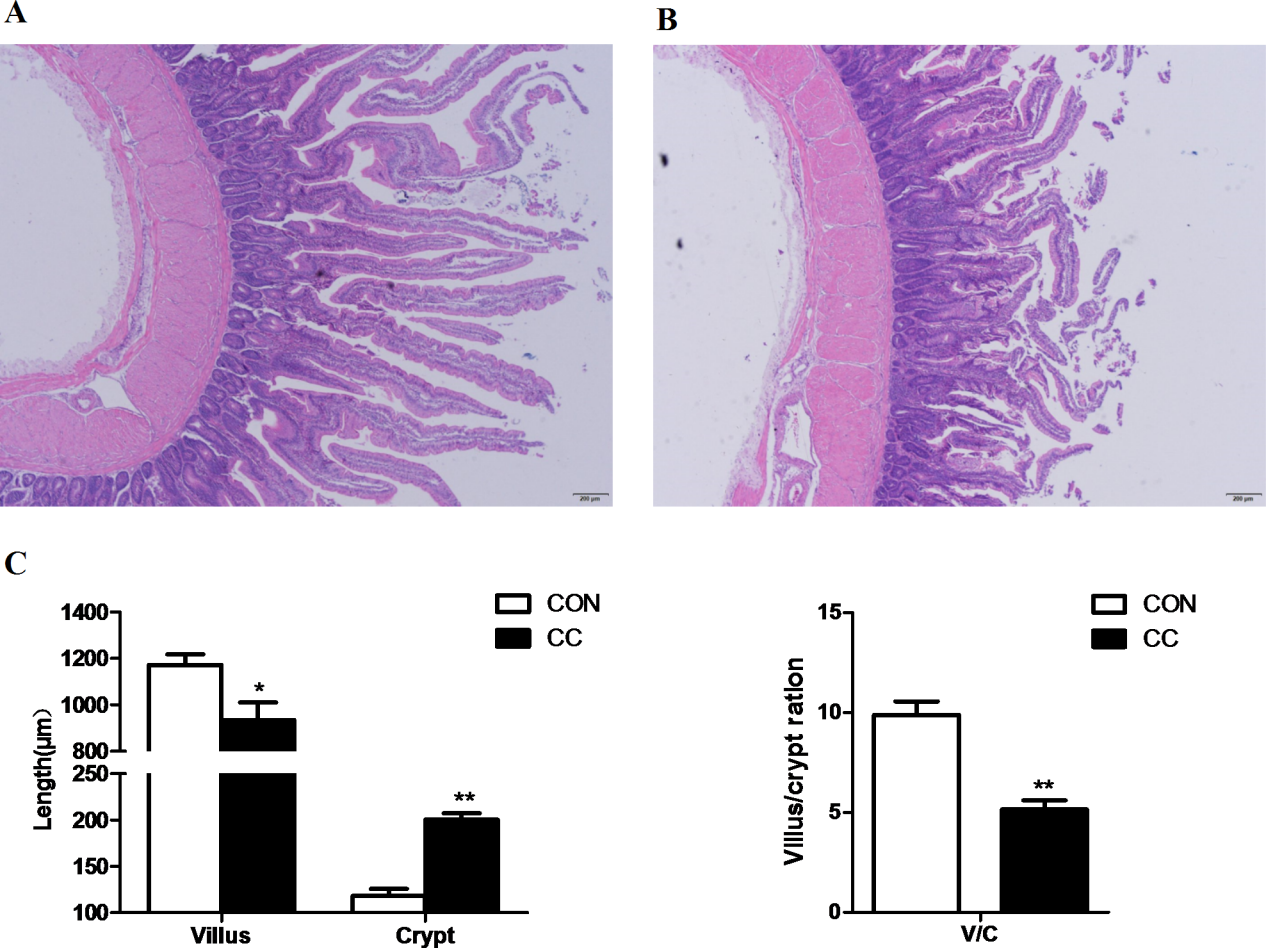
Comparisons of morphological of the duodenum mucosa between CON and CC chickens Duodenum mucosa epithelium (n = 4) from each group were processed for morphological evaluation: duodenum section of CON group (**A.** scale bar = 200 μm); CC group (**B.** scale bar = 200 μm). The relative villus, crypt length and the ration of villus and crypt were measured **C.** * Mean value was significantly different from that of the control group (P < 0.05).

**Figure 2:**
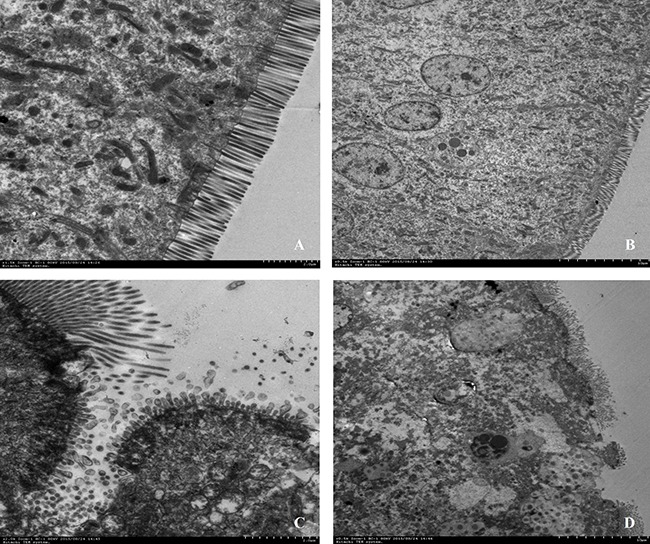
Comparisons of ultrastructure of the duodenum mucosa between CON and CC chickens Duodenum mucosa epithelium (n = 3) from each group were processed for ultrastructure evaluation: duodenum section of CON group **A.** and **B.** duodenum section of CC group **C.** and **D.** (A and C, Scale bar = 2μm; B and D, Scale bar = 10μm).

HE staining showed that severe cellular damages, indentations and villus fracture were observed in the duodenum epithelium of CC chickens but not in CON. CC chickens had longer depth of crypts (P < 0.01), but lower height of villus (P = 0.01) compared to CON counterparts; therefore, the ratio of the length of villus to crypt was significantly lower in CC birds than CON (P < 0.01) (Figure 1). Moreover, the ultrastructure of the duodenum epithelium was detected by the transmission electron microscopic (TEM) method. CC birds displayed swollen villis, wider intercellular space and apparent nuclear breakdown, while the control chickens exhibited integrity and normal epithelia morphology and structure of duodenum (Figure 2).

### Plasma corticosterone concentration and Tch content in plasma and the duodenum mucosa

**Figure 3:**
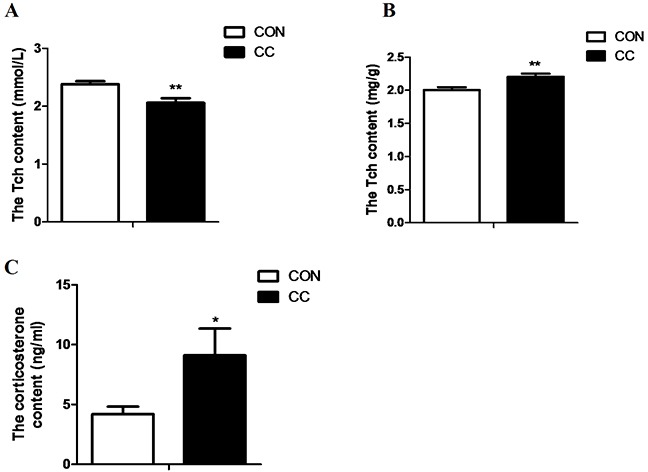
Comparisons of cholesterol content in plasma and the duodenum epithelium and plasma corticosterone between CON and CC chickens **A.** cholesterol content in serum. **B.** cholesterol content in the duodenum epithelium. **C.** corticorsterone content in serum. Data are presented as means {plus minus} SEM. n =14/group. * Mean value was significantly different from that of the control group (P < 0.05).

The results showed that the level of total cholesterol (Tch) in plasma was significantly reduced by CC infection (P < 0.01), while the content of Tch in local duodenum mucosa (P < 0.05) was markedly increased in CC chickens compared to CON. Stress hormone corticosterone concentration in plasma was doubled in CC chickens and was significantly higher than that in CON (P < 0.05) (Figure 3).

### Expression of detected innate immune genes in the duodenum epithelium

**Figure 4:**
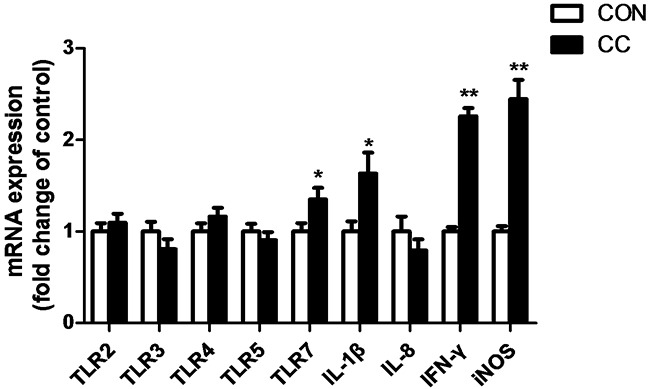
Comparisons of mRNA expression of inflammatory factors in the duodenum between CON and CC chickens Con = control; CC = Coccidial challenge. Data are expressed by means ± SEM. n =12/group. * Mean value was significantly different from that of the control group (P < 0.05).

The abundance of mRNAs encoding innate immune genes and genes involved in inflammatory response were greatly up-regulated in the duodenum epithelium of CC group compared to CON (Figure 4). Coccidiosis infection significantly increased TLR7 mRNA expression (P < 0.05) but not other TLRs, and enhanced interleukin-1β (IL-1β) (P < 0.05) and interferon γ (IFN-γ) (P < 0.01) as well as inducible nitric oxide synthetase (iNOS) mRNA expression (P < 0.01) in the duodenum epithelium.

### Changes of cell proliferation in the duodenum epithelium

**Figure 5:**
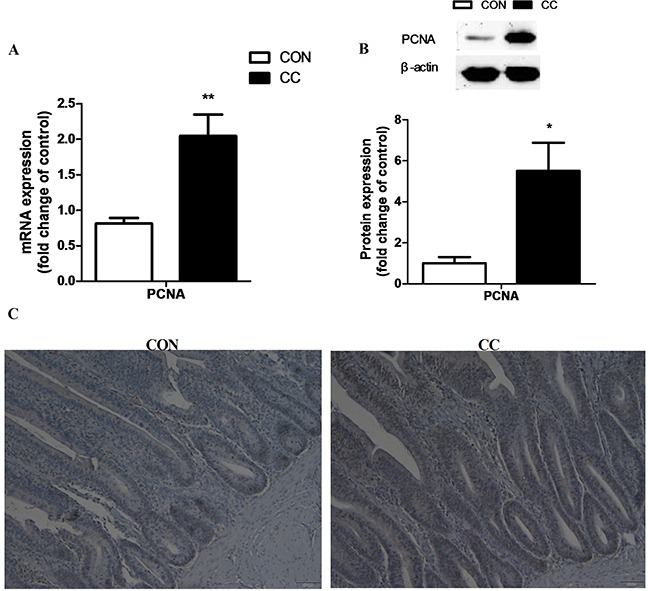
Effects of coccidial infection on the mRNA and protein levels of proliferating cell nuclear antigen (PCNA) in the duodenum mucosa **A.** mRNA expression, n = 12/group. **B.** protein expression, n = 9/group. Con = control; CC = Coccidial challenge. Data are expressed by means ± SEM. * Mean value was significantly different from that of the control group (P < 0.05). Serial sections of chicken duodenum stained with PCNA IHC: duodenum section of the CON and CC group (**C.** scale bar = 50 μm).

In order to elucidate whether the increase of cholesterol contributes to the division of stem cells in the duodenum epithelium, cell proliferative marker proliferating cell nuclear antigen (PCNA) mRNA and protein expression were detected by real-time PCR, western blot and immunohistochemical (IHC) analysis, respectively (Figure 5). Results showed that the abundance of PCNA mRNA (P < 0.01) and protein (P < 0.05) in the duodenum epithelium of CC group was significantly up-regulated compared to CON. The number of PCNA positive signal cells was also increased in the duodenum epithelium by coccidiosis-challenged.

### Alterations of mRNA, miRNA and protein expression of key factors involved in cholesterol metabolism in the duodenum epithelium of CON and CC groups

**Figure 6:**
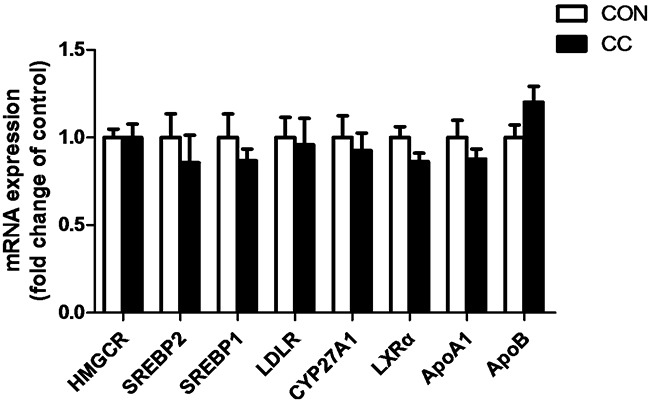
Effects of coccidial infection on genes expression involved in cholesterol metabolism in the duodenum mucosa Con = control; CC = Coccidial challenge. Data are expressed by means ± SEM. n =12/group.

**Figure 7:**
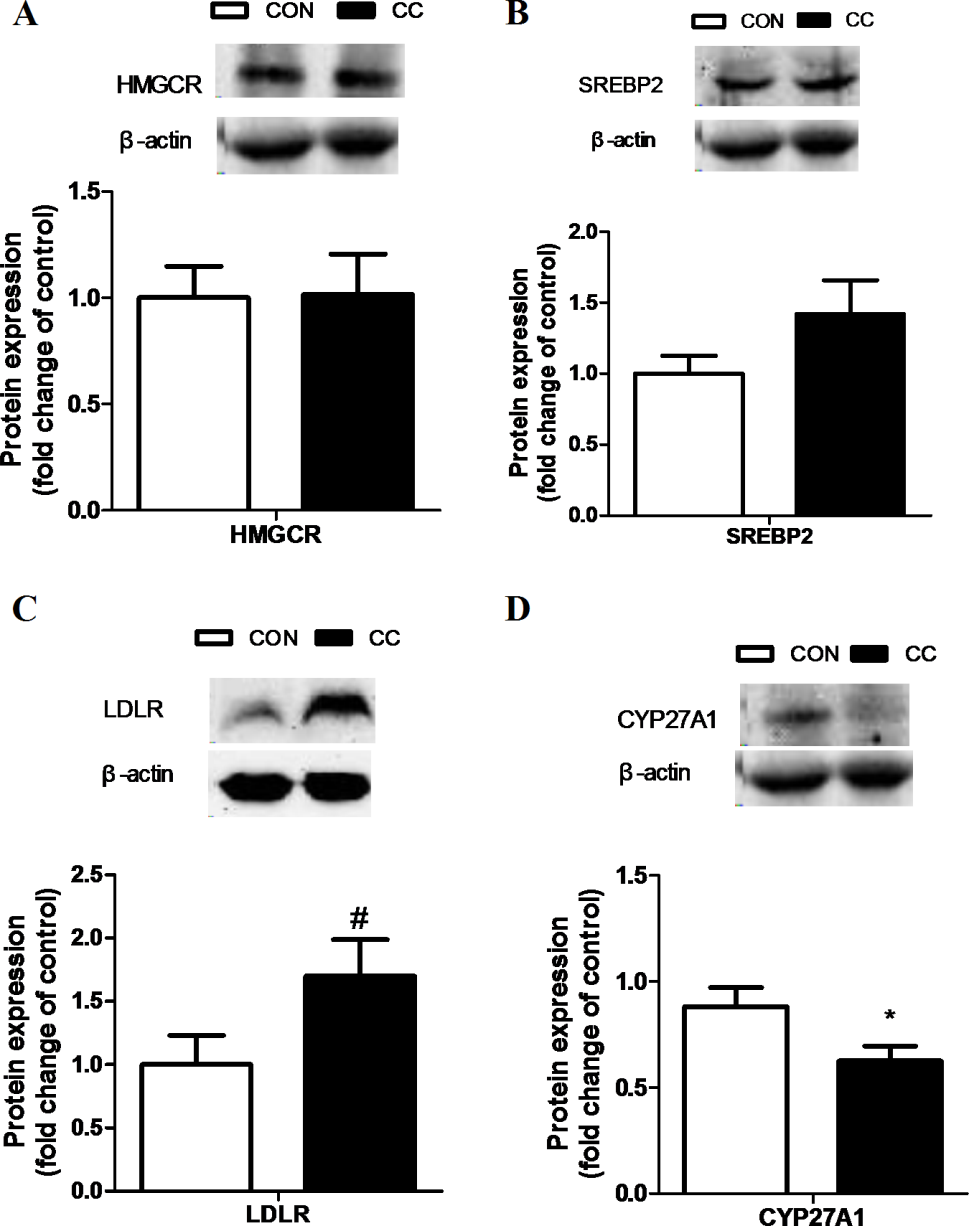
Effects of coccidial infection on proteins expression involved in cholesterol metabolism in the duodenum mucosa Protein expression of the 3-hydroxy-3-methylglutaryl CoA reductase (HMGCR) **A.** the sterol regulatory element binding protein 2 (SREBP-2) **B.** the LDL receptor (LDLR) **C.** and the cholesterol-27a-hydroxylase (CYP27A1) **D.** Con = control; CC = Coccidial challenge. Data are expressed by means ± SEM. n =9/group. * Mean value was significantly different from that of the control group (P < 0.05).

**Figure 8:**
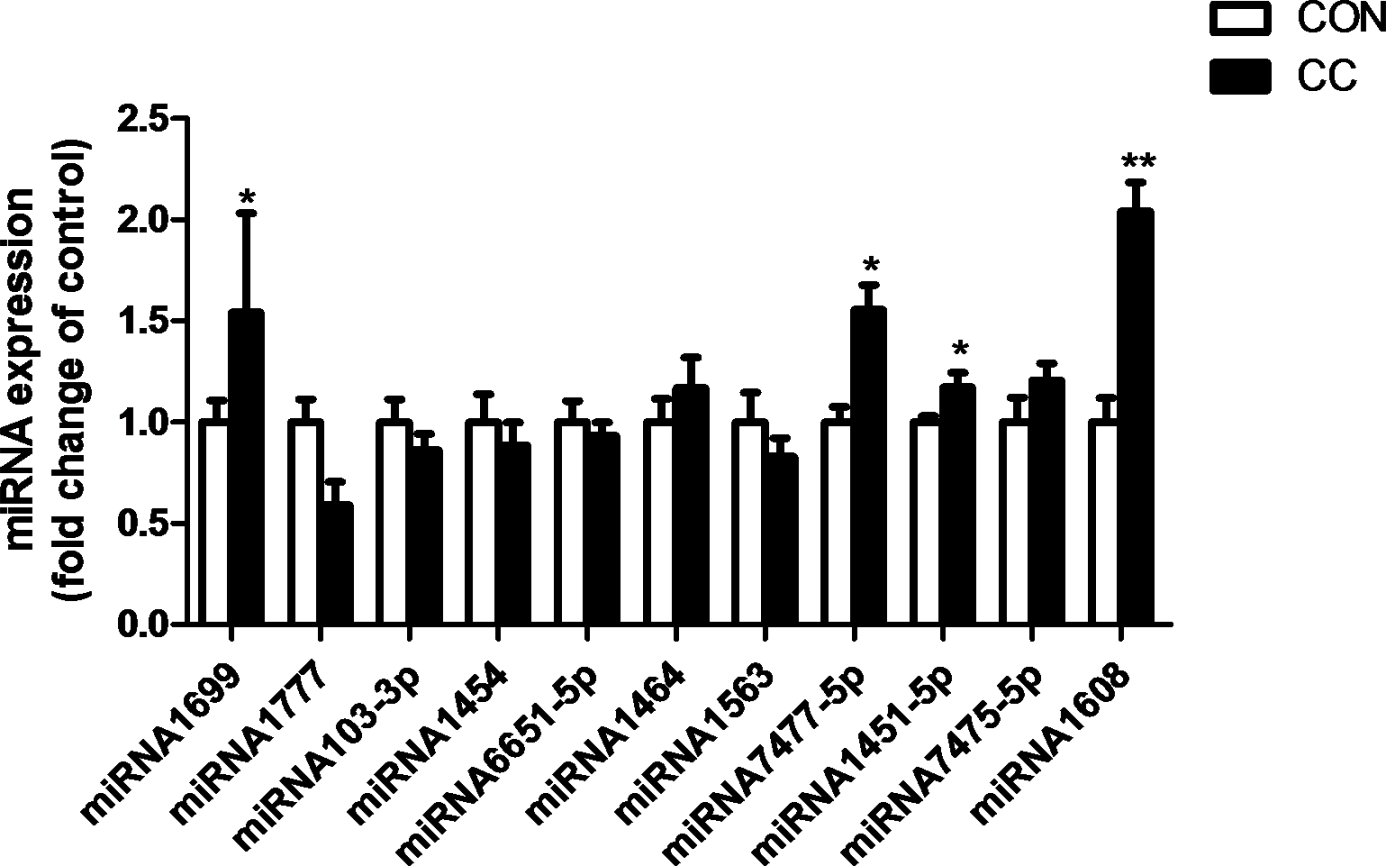
Effects of coccidial infection on miRNAs expression predicted to target cholesterol-27a-hydroxylase (CYP27A1) in the duodenum mucosa Con = control; CC = Coccidial challenge. Data are expressed by means ± SEM. n =10/group. * Mean value was significantly different from that of the control group (P < 0.05).

There was no significant difference of genes expression including 3-hydroxy-3-methylglutaryl-CoA reductase (HMGCR), sterol regulatory element binding protein 1 and 2 (SREBP 1 and 2), sterol 27-hydroxylase (CYP27A1), low density lipoprotein receptor (LDLR), liver x receptor alpha (LXRα), apolipoproteins A1 and B (APOA1 and APOB) in the duodenum epithelium between two groups (P > 0.05) (Figure 6). However, compared to CON, the intensity of LDLR protein showed a tendency to increase in CC group (P = 0.08), while the abundance of CYP27A1 protein was markedly decreased in the duodenum by coccidial-challenged (P < 0.05) (Figure 7). The discrepancy between CYP27A1 mRNA and protein expression indicates the post-transcriptional regulation in the duodenum epithelium of CC group. Therefore, the level of microRNAs (miRNAs) specifically targeting to CYP27A1 mRNA was measured by real-time PCR. Among eleven miRNAs, four miRNAs including miR-1699, miR-7477-5p, miR-1451-5p and miR-1608 expression were significantly increased in CC group compared to CON (P < 0.05), which was consistent with the down-regulation of CYP27A1 protein expression (Figure 8).

### Functional validation of miR-1608 and miR-1699

**Figure 9:**
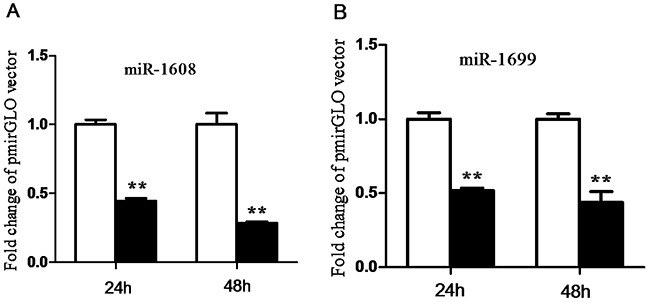
Validation of miR-1608 and miR-1699 targeting cholesterol-27a-hydroxylase (CYP27A1) 3′-UTR **A.** □, pmirGLO vector; ■, pmirGLO-CYP27A1-3′UTR vector. **B.** □, pmirGLO vector; ■, pmirGLO-CYP27A1-3′UTR vector. Data are expressed by means ± SEM. n =3/group. * Mean value was significantly different from that of the control group (P < 0.05).

In order to investigate the functional validation of miR-1608 and miR-1699, a luciferase reporter system was conducted in this study. We found that ectopic expression of miR-1608 and miR-1699 significantly suppressed (p < 0.05) the luciferase activity of HeLa cells co-transfected with the pmirGLO-CYP27A1-3′UTR dual-luciferase reporter plasmid at both 24 and 48 h (Figure 9).

## DISCUSSION

Coccidial infection is a common event occurred in most mammals and avian species that typically leads to the intestine structural disruption and physiological dysfunction [18, 19]. In this study, we also found severe structural damages in the duodenum of chickens infected with E. Acervulina as observed in the previous studies [20, 21]. Local inflammation was also confirmed by the increase of TLR-7, the pro-inflammatory cytokine IL-1β and INF-γ genes expression involved in the innate immune response. Moreover, the stress hormone corticosterone concentration in the circulating system was greatly increased in CC chickens indicating a high stress response induced by coccidial infection. Consequently, chickens infected with coccidial displayed lower growth performance with higher food intake but lower feed conversion efficiency as previous reports [5, 22].

The pro-inflammatory molecules induce inflammation and participate in epithelial repair and healing processes through recruitment and activation of immune cells in the intestine [9]. It's suggested that cytokines might contribute to restoring epithelial barrier integrity by inducing controlled proliferation of stem cells at the crypts during the acute phase of inflammation. However, during the chronic inflammation these molecules such us IL-18 and IFN-γ might inhibit epithelial cell proliferation in neoplastic regions of the intestine [10]. In this study, an increase of cell proliferation was also observed in the crypt base of coccidia-infected chickens, which exhibited higher length of the crypts and higher PCNA signals in the duodenum epithelium. The abundance of PCNA signals represents the total mitotic cells present in the mucosa, including the epithelial population and lamina propria cells. As in mammals, the intestinal integrity in birds is regulated tightly by many different signaling pathways which balance cell proliferation and differentiation along the crypt-villus axis [23, 24].

The availability of cholesterol is a prerequisite for cell proliferation and cellular growth, and change in membrane cholesterol content in the intestinal mucosa may affect membrane functions during development. It's documented that cholesterol is enriched in membrane microdomains perhaps mediating signal transduction [25]. A disruption in microdomain structures caused by reduced cholesterol content may prevent endocytosis of toxins or invasion by bacteria [26]. Evidences also suggest that local cholesterol synthesis in the gut is to support the rapid enterocyte proliferation [27]. Consistently, our results showed that local content of cholesterol was significantly increased in duodenum after coccidia-infection. On the contrary, the level of systemic cholesterol was greatly decreased in CC chickens, which might be due to the significant increase of corticosterone production. It's well known that cholesterol is the precursor for glucocorticoids and sexual hormones production in mammals and birds. The discrepancy between circulating and local cholesterol content in intestine indicates that the alterations of local cholesterol metabolism are responsible for its accumulation in the duodenum epithelia in CC chickens.

The small intestine is quantitatively the most important organ involving in both cholesterol synthesis and LDL cholesterol uptake [27]. LDLR complexes are present in the cell surface mainly participating in an internalized process known as endocytosis. This process occurs in the majority of body cells including the epithelial cells in the small intestine. In this study, we found a moderate increase of LDLR protein in the duodenum epithelium of the coccidial-challenged chickens, which might contribute at least partially to the increase of local cholesterol content. Moreover, a significant decrease of CYP27A1 protein in the duodenum epithelium was observed in CC chickens compared to control. It's well known that cholesterol can be catabolized into bile acids through CYP27A1 and CYP7A1 in the liver [28–30]. The intestine is exposed to very high levels of dietary cholesterol, which may activate CYP27A1 in intestine to regulate intestinal cholesterol efflux [15]. The activation of pregnane x receptor (PXR) and CYP27A1 may be an adaptive response to a high-cholesterol diet to remove excessive un-esterified cholesterol in the intestine [31]. Taken together, our results suggest that the decrease of CYP27A1 protein combined with a moderate increase of LDLR may lead to intestinal cholesterol accumulation, and the latter is to support the epithelial cells proliferation during the inflammation induced by coccidial infection.

In the present study genes involved in cholesterol metabolism were not altered at the transcriptional level. The dissociation of mRNA and protein expression of CYP27A1 indicates the post-transcriptional regulation in the intestine. MicroRNAs are well known to participate in the post-transcriptional regulation through targeting mRNA degradation and/or translation repression [32]. When transcriptional regulation and miRNA-mediated translational repression are not synchronized, mRNA and protein levels can be uncoupled. In this study, the expression of four miRNAs targeting to CYP27A1 including miR-1699, miR-7477-5p, miR-1451-5p and miR-1608 was significantly increased in coccidia-infected chickens, which was associated with decreased protein content of CYP27A1 in the duodenum epithelium. To verify the targeting sites of miR-1608 and miR-1699 on 3′-UTR of CYP27A1 transcripts, we conducted luciferase reporter gene assay and confirmed that miR-1608 and miR-1699 inhibited luciferase activity through targeting 3′-UTR of CYP27A1. However, the nature behind the increase of miRNAs expression still needs further study.

It's very important to note that besides the similarity of histological features of intestine epithelia between avian and mammals, cell proliferation mainly occurs in the stem cell zone located in the intestinal crypt. Intestinal homeostasis in chicken is also assumed to be similar to that of mammals [33]. Moreover, chickens are a good model for studying the disorders of cholesterol metabolism involved in the development of spontaneous arterial atherosclerotic lesions in human [34]. Therefore, it's reasonable to speculate that the conclusion derived from the current study has a potential impact on cancer patients in clinic.

In conclusion, we demonstrated here for the first time that E. acervulina infection induced intestinal inflammation, epithelial proliferation and the increase of cholesterol accumulation in duodenum epithelia. The increase of LDLR combined with the decrease of CYP27A1 protein may be at least partially responsible for local cholesterol accumulation, which contributes to the epithelial proliferation induced by infection. Moreover, the post-transcriptional regulation of miRNAs targeting to CYP27A1 was involved in this process. Taken together, our results provide a new insight for interventions of epithelial proliferation and cholesterol metabolism in the gastrointestinal tracts.

## MATERIALS AND METHODS

### Animals and experimental design

One hundred white-feather broilers (a local commercial breed) were purchased from a commercial hatchery, then were wing-banded and housed in the brood chamber. House temperature was maintained at 34 ± 3°C in the first 3 d, and decreased gradually to 21 ± 3°C at 28 d of age. Starter crumble was provided from 1 d to 20 d, and then finisher pellet was provided from 21 d to 39 d. Continuous lighting was conducted during the first week, and the lighting time decreased gradually by 2 h/week to 18 L:6 D at 21 d, and kept constant to 39 d. On 32 d, forty chickens with body weight close to the mean value in the whole group were caged and divided randomly into two groups. The remaining chickens were excluded from the study. All birds had free access to feed and water during the whole experimental period.

All animal procedures were approved by the Institutional Animal Care and Use Committee of Nanjing Agricultural University. The protocol of this study was reviewed and approved specifically, with the project number 2011CB100802. The slaughter and sampling procedures strictly followed the ‘Guidelines on Ethical Treatment of Experimental Animals’ (2006) no. 398 set by the Ministry of Science and Technology, China and the ‘Regulation regarding the Management and Treatment of Experimental Animals’ (2008) no. 45 set by the Jiangsu Provincial People's Government.

### Coccidial infection

At 32 d of age, chickens in the coccidiosis-challenged groups (CC) were challenged with E. acervulina (200.000 oocysts/chicken, n = 20) by oral administration and the unchallenged groups received the diluents as the control (CON). Body weight and feed intake (FI) per pen were recorded during the experiment.

### Blood and tissue sampling

Chickens which were randomly selected from each replicate were weighed and killed by I.V. injection of pentobarbital (30-40 mg/kg) for tissue sampling on the 39 d. Blood samples were collected and then centrifuge (3500 rpm, 10 min), the serum samples were stored at -20°C. The duodenum samples were collected into liquid nitrogen and then stored at -70°C for subsequent RNA and protein extraction.

### Morphological analysis

Specimens of the duodenum were prepared for histological examination by fixing in 4% formaldehyde-buffered solution, embedding in paraffin, and sectioning (5 μm). Specimens were examined for injury after hematoxylin and eosin (H&E) staining as described by Makishima et al [35]. A total of 10 intact, well-oriented villus–crypt units were selected for each intestinal cross section. Villus height (μm) was measured from the tip of the villus to the villus–crypt duodenum, and crypt depth was measured from the base upward to the region of transition between the crypt and villus.

Duodenum mucosa tissue samples were separated and fixed immediately with 2% glutaraldehyde, post-fixed with 1% osmium tetroxide, and embedded in resin. Ultrathin sections were cut and stained with uranyl acetate and lead citrate. Epithelial tissues ultrastructure was determined with a transmission electron microscope (Hitachi H-7650, Hitachi Technologies, Tokyo, Japan).

### Immunohistochemical analysis (IHC)

Immunohistochemical detection of proliferating cell nuclear antigen (PCNA) was performed on the duodenum sections as described previously [36]. In brief, representative paraffin sections (5 μm) were subjected to immunohistochemistry staining. Tissue sections were quenched for endogenous peroxidase (0.01 M citrate buffer, pH 6.0) for 15 min in a water bath at 97°C. Then, after incubation in 5 % BSA for 30 min at 37°C, mouse MAb anti-PCNA (clone PC10; Santa) was applied to the sections at dilutions of 1:50 for overnight at 4°C. After washing, slides were stained with a biotin-conjugated secondary antibody, followed by Streptavidin-Biotin complex (SA1051, Boster Company, Nanjing, China) for 30 min each at 37°C. Staining was developed with DAB (AR1000, Boster Company, Nanjing, China), slides were counterstained with hematoxylin, dehydrated, and mounted. For negative control in the IHC procedures performed, PBS replaced the primary antibodies.

### Measurement of cholesterol in serum and tissue, corticosterone in serum

One hundred milligram frozen duodenum was minced and homogenized by crushing equipment (MS-100R; Japan) in 1 mL of ice-cold homogenization buffer RIPA (Pulilai, Beijing). The homogenate was centrifuged at 12,000 rpm for 20 min at 4°C and then collected the supernatant. The cholesterol (CHOL) was measured using commercial kits (Pulilai, Beijing, China) according to the instruction provided by the manufacturer. The serum concentration of cholesterol (CHOL) were measured by automatic biochemical analyzer (Tokyo Japan, Hitachi7020) using commercial kits (Jiancheng Bioengineering Institute, Nanjing, China). CORT concentration in serum was measured using a commercial EIA kit (No. 500655, Cayman, USA) according to the instructions of the manufacturer.

### RNA, cDNA synthesis and real-time PCR

Total RNA was extracted from duodenum samples with Trizol Reagent (Cat#3101-100, Pufei, Shanghai, China). Concentration of the RNA was measured by NanoDrop ND-1000 Spectrophotometer (Thermo Fisher, USA). The ratios of absorption (260/280 nm) of all samples were between 1.9 and 2.0. The quality of RNA was assessed by electrophoresis on an agarose gel. 500 nanogram of total RNA was reverse-transcribed according to manufacturer's instructions (Vazyme Biotech, Nanjing, China). 2 uL of diluted cDNA (1:25, vol/vol) was used for real-time PCR which was detected in Mx3000P (Stratagene, USA). TBP was used as a reference gene [37]. All the primers synthesized by Generay (Shanghai, China) were listed in Table 2. The method of 2^−ΔΔ^Ct was used to analyze the real-time PCR results, and gene mRNA expression was showed as the fold change relative to the mean value of control group [28].

**Table 2:** Nucleotide sequences of specific primers

Gene	Sequence 5′–3′	GenBank accession no.
TBP	ATAGTGCCACAGCTACAGA	NM_205103.1
	GTACGTGGTTCTCTTATCCTC	
HMGCR	TTGGATAGAGGGAAGAGGGAAG	NM_204485.1
	CCATAGCAGAACCCACCAGA	
SREBP1	CTACCGCTCATCCATCAACG	AY029224
	CTGCTTCAGCTTCTGGTTGC	
SREBP2	CCCAGAACAGCAAGCAAGG	XM_416222
	GCGAGGACAGGAAAGAGAGTG	
CYP27A1	AGGACTTTCGTCTGGCTCT	XM422056.4
	CTCCGCATCGGGTATTT	
LDLR	CCACCATTTGGCAGAGGAA	NM_204452.1
	ACCGCAGTCAGACCAGAAGAG	
ApoA1	GTGACCCTCGCTGTGCTCTT	NM_205525.4
	CACTCAGCGTGTCCAGGTTGT	
ApoB	GCATCTCTGCATCTCAGGAAAGA	NM_001044633.1
	GCAGGCTACAAACTAACAGATCCA	
LXRα	GCAACTACCTGGCTTCCGAGA	AJ507202.1
	CTGCTTTGGCGAAGTCATCCC	
PCNA	TGAATGAGCCAGTCCAG	NM_204170.2
	AGTGTCCCATATCAGCAA	
TLR2	ATCCTGCTGGAGCCCATTCAGAG	NM_204278.1
	TTGCTCTTCATCAGGAGGCCACTC	
TLR3	TCAGTACATTTGTAACACCCCGCC	NM_001011691
	GGCGTCATAATCAAACACTCC	
TLR4	TGCCATCCCAACCCAACCACAG	AY064697
	ACACCCACTGAGCAGCACCAA	
TLR5	GCCTGGGGAAGAACATATCAAC	AJ626848
	GGCTTCTACACACCACCCATC	
TLR7	GGCTGTGAATGAATGGGTGA	NM_001011688
	GCTGAATGCTCTGGGAAAGG	
IL-1β	GTGAGGCTAACATTGCGCTGTA	Y15006.1
	TGTCCAGGCGGTAGAAGATGAAG	
IL-8	TCCTGGTTTCAGCTGCTCTGT	NM_205498
	CGCAGCTCATTCCCCATCT	
IFN-γ	ACACTGACAAGTCAAAGCCGCACA	X99774
	AGTCGTTCATCGGGAGCTTGGC	
iNOS	AATGCTGTGCCCATGGCAGTTTGCA	D85422
	CACCTCAAGGAGCATGTTGGCAACA	

### Protein extraction and western blot analysis

One hundred milligram of frozen duodenum epithelium was minced and homogenized by crushing equipment (MS-100R; Japan) in 0.8 mL of ice-cold homogenization buffer RIPA containing the protease inhibitor cocktail Complete EDTA-free and PhosSTOP (Roche, Germany). The homogenate was centrifuged at 12,000 rpm for 20 min at 4°C and then collected the supernatant. Protein concentration was measured using a BCA Protein Assay kit (Pierce, Rockford, USA). 40 micrograms of protein extracted from each sample was loaded onto 10% SDA-PAGE gel. Into nitrocellulose membranes (Bio Trace, Pall Co., USA) and blocked for 2 h at room temperature, then were incubated with the following primary antibodies: HMGCR (BS6625, Bioworld Technology, 1:500), SREBP2 (SC-5603,Santa cruz; USA; 1:500), CYP27A1 (BS2192, Bioworld Technology, 1:500), LDLR (10785-1, proteintech, 1:500), PCNA (SC-56, Santa, 1:500), β-actin (AP0060, Bioworld, USA; 1:10,000), in dilution buffer for one night at 4°C. After three times (every 10 min) washes in Tris-buffered-saline with Tween (TBST), membranes were incubated with goat anti-rabbit horseradish peroxidase (HRP)-conjugated secondary antibodies (Bioworld, USA; 1:10,000) in dilution buffer for 2 h at room temperature. After three times (every 10 min) washes, bands were visualized by enhanced chemiluminescence's (ECL) using the LumiClo substrate (Super SignalWest Pico Trial Kit, Pierce, USA), signals were recorded and analyzed respectively by an imaging system (Bio-Rad, USA) and Quantity One software (Bio-Rad, USA). Values of protein were presented as fold change relative to the average value of CON group.

### MiRNAs expression assay

Total RNA (2 ug) treated with RNase-free DNase I (Promega, USA) were polyadenylated by poly(A) polymerase using a poly(A) tailing kit (AM1350; Applied Biosystems), according to the manufacturer's instructions. Polyadenylated RNA was then dissolved and reverse transcribed using a poly(T) adapter. Realtime PCR was performed with SYBR Green qPCR master mix reagent (Takara) in triplicate using a microRNA (miRNA)-specific forward primer and a universal reverse primer complementary to part of the poly(T) adapter sequence.U6 small-nuclear RNA (U6 snRNA) was used as a reference gene to normalise the expression of miRNA [38]. The sequences of all porcine miRNA were acquired from miRBase (http://www.mirbase.org/). miRNA targeting CYP27A1were predicted with an online miRNA prediction tool [39]. Among all the predicted miRNA, eleven targeting CYP27A1were quantified by real-time PCR. The primer sequences used for miRNA analysis are listed in Table 3.

**Table 3:** miRNA and the corresponding primer sequences

Names	Primer sequences	miRbase Accession
gga-miR-1699	CCAGAGGGACATGGCAGGGCAA	MIMAT0007591
gga-miR-1777	GTGGGCGGTGCGGGGCGGCG	MIMAT0007688
gga-miR-103-3p	AGCAGCATTGTACAGGGCTATGA	MIMAT0001145
gga-miR-1454	GTACAATGATGAGACTTTGGCTCC	MIMAT0007331
gga-miR-6651-5p	ACCAGGTTGCCTAAGGAGGGG	MIMAT0025751
gga-miR-1464	TGCTGTTTGCAGGGCCGCCTCGGA	MIMAT0007342
gga-miR-1563	GCACATGATGAGGAAGCACTGAAA	MIMAT0007422
gga-miR-7477-5p	ACAGCGCGGATCGGGGCTGGGAG	MIMAT0029108
gga-miR-1451-5p	TCGCACAGGAGCAAGTTACCGC	MIMAT0007324
gga-miR-7475-5p	CCGCCGCCGCCGCGCCCTCC	MIMAT0029104
gga-miR-1608	TGGGACAGTGGCTGCGCCCTCT	MIMAT0007474
universal reverse primer	TAGAGTGAGTGTAGCGAGCA	N/A
poly(T) adapter	TAGAGTGAGTGTAGCGAGCACAGA	N/A
	ATTAATACGACTCACTATAGG(T)16VN	
U6	CTCGCTTCGGCAGCACATATACT	N/A

### Co-transfections and dual luciferase activity assay

The pmirGLO-CYP27A1-3′UTR vector was constructed, by inserting CYP27A1 3′UTR with the putative binding site of miR-1608 and site of miR-1699 at the downstream of the firefly luciferase gene into pmir vector (Promega). Plasmids were prepared with E.Z.N.A.^TM^ Endo-free Plasmid Maxi Kit (Omega Bio-tek). HeLa cells (1×10^5^ cells per well) were seeded in a 24-well culture plate and cultured with DMEM medium with 10% fetal bovine serum (FBS). As the confluence of HeLa cells reached 80-90%, pmirGLO vector (174 ng) and pmirGLO-CYP27A1-3′UTR vector (174 ng) were transfected into HeLa cells simultaneously with 100 nM miRNA mimics via Lipofectamine 2000TM (Invitrogen). Twenty-four hours and forty-eight hours later, the luciferase activity was detected according to the manufacturer's instruction using the luciferase reporter assay system (GloMax® 96 Microplate Luminometer; Promega), and the relative luciferase activity value showed as FL/RL that was achieved from the firefly luciferase activity by the Renilla luciferase as control per sample. The normalized relative luciferase activity of different groups was used for statistical analysis.

### Statistical analysis

Data are presented as means ± SEM. The data were tested for normal distribution, and statistical significance was assessed by Student's unpaired test using the software package SPSS version 18.0 for Windows (SPSS Inc., Chicago, IL, USA). Data were considered statistically significant when P < 0.05.
